# Unlocking the potential: Key factors shaping the liquid biofuels market in Ukraine

**DOI:** 10.1016/j.heliyon.2024.e40420

**Published:** 2024-11-15

**Authors:** Galyna Trypolska

**Affiliations:** Institute for Economics and Forecasting of the National Academy of Sciences of Ukraine, P.Myrnogo str., 26, Kyiv, 01011, Ukraine

**Keywords:** Biofuels, Bioethanol, Biodiesel, Ukraine

## Abstract

This article examines the development of Ukraine's liquid biofuels market, driven by the need to diversify its fuel supply amidst complete reliance on imported fuels for road transport. The country also faces challenges exporting agricultural feedstock for first-generation biofuels due to geopolitical disruptions since early 2022. In response, the production and use of liquid biofuels represent a strategic move to reduce dependence on fossil fuels while fulfilling international obligations, including the ones within the Energy Community and the European Union (EU). Ukraine must align with the EU's renewable energy targets as a candidate for EU accession. Using the methods of fuel consumption quantification, blending mandate assessment, and excise tax rate calculation framework, the article finds that the potential market demand for bioethanol in Ukraine is projected to range from 125 to 278 thousand tonnes and for biodiesel from 196 to 431 thousand tonnes in 2025–2031. Ukraine's biofuel production potential could reach 2.54 million tonnes of oil equivalent by 2050, with advanced biofuels expected to dominate. Current excise tax rates for bioethanol are considered appropriate and supportive of market growth. To foster further development, legislative amendments are needed to mandate biodiesel blending, along with awareness campaigns highlighting the benefits of biofuels. Additionally, vehicles using high biofuel blends should receive incentives similar to those of electric vehicles, such as access to preferential parking.

## Highlights

• Ukraine's bioethanol market demand may reach 278 thousand tons by 2031.
• Biofuel production could reach 2.54 million t o.e. by 2050 in Ukraine.
• Ukraine's current excise tax rates for bioethanol support biofuel market growth.

## Abbreviations:

cm^3^ –: cubic centimeter
CO_2_-eq -: CO_2_-equivalent
ETBE –: Ethyl Tert-Butyl Ether
EU –: European Union
EUR –: Euro
FAEAE –: Fatty Acid Ethyl Ester
FAMAE –: Fatty Acid Methyl Ester
FAO –: Food and Agriculture Organization
g –: gram
GDP –: Gross Domestic Product
GHG –: Greenhouse Gases
ha -: hectares
HVO –: Hydrotreated Vegetable Oil
ILUC –: Indirect Land Use Change
kg -: kilogram
kton –: 1000 tonnes
L –: liter
m^3^ –: cubic meter
MJ –: Megajoule
Mt –: Megatonne
MTBE –: Methyl Tert-Butyl Ether
RED II –: Renewable Energy Directive II
t o.e –: tonne(s) of oil equivalent
UAH –: Ukrainian Hryvnia
UNECE –: United Nations Economic Commission for Europe
USA –: the United States of America
USD –: United States Dollar
VAT –: Value Added Tax

## 1 Introduction

Meeting energy needs is a challenge for many countries globally. With the growing understanding of environmental problems and the need to combat climate change, governments worldwide are actively implementing alternative energy sources and continuously conducting scientific research to find new, more efficient energy carriers. Liquid biofuels in various forms have several advantages. Still, the primary and most universal argument for its use is the attempt to reduce dependence on fossil fuels, which are often imported.

According to current Ukrainian legislation, several types of liquid biofuels are distinguished. Bioethanol is dehydrated ethyl alcohol made from biomass or crude ethyl alcohol for use as biofuel or a biocomponent [1]. Alternative fuels are solid, liquid, or gaseous energy sources that replace conventional fuels. They are derived from non-traditional raw materials or energy sources that differ from those typically used in conventional fuel production [2]. Alternative transport fuels contain 30 % or more of the biological component. Biodiesel fuel (biodiesel) consists of methyl and/or ethyl esters of higher organic acids obtained from vegetable oils or animal fats, used as biofuel or a biocomponent [3]. Hydrotreated Vegetable Oil is renewable diesel fuels derived from various feedstocks such as tall oil, rapeseed oil, waste cooking oil [4]. Bioethanol-based additives are motor fuel biocomponents obtained by synthesis using bioethanol or by mixing bioethanol with organic compounds and fuels derived from hydrocarbon raw materials [5]. Specifically, this refers to mixtures of ethyl alcohol used as feedstock for the production of motor vehicle fuels, with an ethyl alcohol concentration of 50 % by volume or more. Additionally, there can be bioethanol with a concentration of denatured ethyl alcohol of 97.8 % by volume or more, which will be used by companies solely for the production of bioethanol, motor gasoline blends containing bioethanol, and other bioethanol-based additives.

It should be noted that in international literature, the term "ethanol" is predominantly used to denote the biological component of fuel. It significantly simplifies calculations and reporting and provides a clear picture of how much biological fuel component has been consumed/produced, etc [[6], [7], [8]]. In Ukrainian legislation and literature, the term "bioethanol" is used, which, as defined earlier, refers to both the biological component of fuel and fossil fuel mixed with a biological component. This dual interpretation is inconvenient and may allow for less transparent ethanol accounting in Ukraine. For example, in the EU, the term "biogasoline" refers to a mixture of gasoline and ethanol, methanol, Ethyl Tert-Butyl Ether (ETBE), or Methyl Tert-Butyl Ether (MTBE) from biomass [9].

Liquid biofuels can be conditionally divided into several types: 1st generation biofuels and advanced biofuels (comprising of the 2nd generation biofuels having lignocellulosic feedstock or the 3rd generation fuels that have no land requirements, such as macro- and microalgae). The 1st generation biofuels are produced from the feedstock that can be food products for human consumption or livestock. Advanced biofuels can significantly contribute to decreasing GHG emissions [10]. Despite countries-leaders in the deployment of renewables (Brazil, the United States of America (USA), selected countries of the EU, and others), globally, the spread of renewables in the transport sector is only 4 %, and the increment rate needs to be more satisfactory [11].

In light of the ongoing full-scale aggression against Ukraine, the need to replace imported fossil fuels has become increasingly critical for the country. This paper aims to examine the foundational elements necessary for developing a liquid biofuels market in Ukraine. It will also estimate the potential demand for liquid biofuels and determine bioethanol's optimal excise tax rate. By addressing these objectives, the study intends to contribute to Ukraine's energy independence and resilience in the face of external challenges.

## 2 Literature review

With the numerous efforts to mitigate climate change, bioeconomy in general and biofuels, in particular, have taken a significant role in trying to keep the rise of global temperature within the 2 °C threshold [10], providing the sustainable use of biomass. The authors stated that numerous uses of biomass do not involve the irrational use of land and do not lead to other adverse effects. Biofuels have many environmental benefits, are less toxic for human health (compared to fossil fuels), and can meet 27 % of global demand for transport fuel, mainly in sectors where the use of fuel hasn't gained total momentum (aviation, maritime transport, long-haul freight vehicles) [12], contributing to a significant reduction of CO_2_ emissions. Agricultural feedstock, when growing, increases evapotranspiration, promoting local cooling, which, in a way, could be considered a climate change adaptation measure [13].

Biofuels can contribute to people's well-being, primarily in rural areas, where the problem of poverty alleviation may be more acute than in urban areas (due to higher employment opportunities). Biofuels positively impact Gross Domestic Product (GDP) growth and household welfare growth [14].

Biofuels present a promising opportunity to soundly utilize the remainings of the agri-food industry, such as used cooking oils, low-quality grains, and animal by-products. This not only contributes to environmental sustainability but also to significant job creation, particularly in the agriculture sector. The potential for job creation is a compelling reason to support the development of biofuels [15].

Többen et al. [16] studied the impact of the global bioeconomy on the land use. They found that despite the bioeconomy positively impacting GDP, it would anticipate significant land-use changes outside the EU, primarily in Latin America and Asia-Pacific countries. Although biofuels would bring emission-reduction benefits, the latter would be offset by the emissions resulting from land-use changes.

The 1st generation biofuels caused numerous debates due to their significant negative externalities [17], first of all the due to their contribution to deforestation and destruction of other valuable lands. The high demand for food products for biofuel production could have contributed to rising food prices and debates over the use of traditional crops for liquid biofuel production, which was seen as exacerbating the global food crisis. Yet, studies indicate that biofuels did not affect global food prices. At the same time, other factors contributed (growing global population, growing crude oil prices, decreasing areas of arable land) even despite the increase in global food production [18]. The areas under the biofuel crops increased due to the profitability of these crops, leading to deforestation and loss of biodiversity in some countries. The 2nd generation biofuels are only a part of a solution, because they also require land for their output, which means that they indirectly compete for the land which otherwise could have been used for food and fodder production [19]. Yet, they can reach higher productivity and bring environmental benefits [10].

The 1st generation biofuels are widely criticized for insufficient CO_2_ emissions reductions. Therefore, the EU is highly critical of the use of such biofuels. According to Ref. [20], the opportunity costs across Europe of allocating millions of hectares of fertile arable land for biofuel production are enormous. Researchers state that this land can be used much more effectively to mitigate climate change, halt biodiversity loss, and increase global food security. Biofuel production has harmed food security and hindered climate change mitigation. Producing cereal crops for biofuel consumed in Europe requires 9.6 million ha of land, an area larger than Ireland. Using land for biofuel feedstock means it is lost mainly as a natural carbon sink, habitat for endangered species, or food production.

One option is to return the land to nature, restoring natural ecosystems mainly through natural processes. In this case, forests and other vegetation restored on an area equivalent to that currently used only for biofuel crops (5.3 million ha) could absorb 64.7 million tonnes of CO_2_ from the atmosphere - almost twice as much as the officially recorded reduction in CO_2_ emissions due to biofuels replacing fossil fuels (32.9 Mt CO_2_-eq.). It would contribute to the EU's stated goals of increasing carbon sinks in the land sector and achieving real climate benefits, contrary to the overestimated emission reductions from biofuels based on incorrect carbon accounting. Using land for solar energy would also be much more efficient. To produce the same amount of energy, 40 times less land is needed for solar-powered electric vehicles compared to cars on biofuels. Plant-based biofuels do not significantly contribute to climate change mitigation, but they hinder it. In addition to climate benefits, "reviving" millions of hectares of land would be a key measure against the massive loss of species. The EU has set a goal to stop and reverse biodiversity loss in the "Nature Restoration Law" adopted by the European Commission in 2022. Biofuel feedstocks require more than 9 % of Europe's arable land [20]. Considering all the negative externalities of the 1st generation biofuels, the latter's production moves towards the advanced biofuels [16].

Das and Gundimeda [21] studied the expansion of biofuels in developing countries. They raised the essential question of whether the negative externalities of first-generation biofuels can be offset by their positive effects. The authors conclude that the food and land use impacts are primarily influenced by a positive agricultural supply response, productivity growth, the scale of the agricultural export market, and the extent of price transmission.

Different countries should develop the types of biofuels that have the best impact on their economies. For instance, Gunatilake et al. [22] stated that India imports 75 % of crude oil and proved that in India, biodiesel enhances energy security and has a positive impact on rural communities (utilizing marginal agricultural resources), while sugarcane ethanol shows no positive effects.

**Fig. 1:**
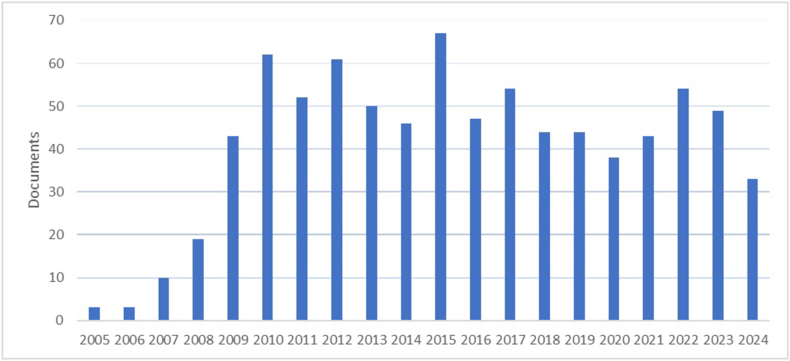
The number of research articles on biofuels, biofuel and energy security in the Scopus database [23].

Overall, biofuels attract the attention of researchers. In the Scopus database alone, the inquiry keywords "biofuels", "biofuel" and "energy security" yielded 822 articles from 2005 to 2024 (Fig. 1).

### 2.1 Potential of liquid biofuels in Ukraine

**Fig. 2:**
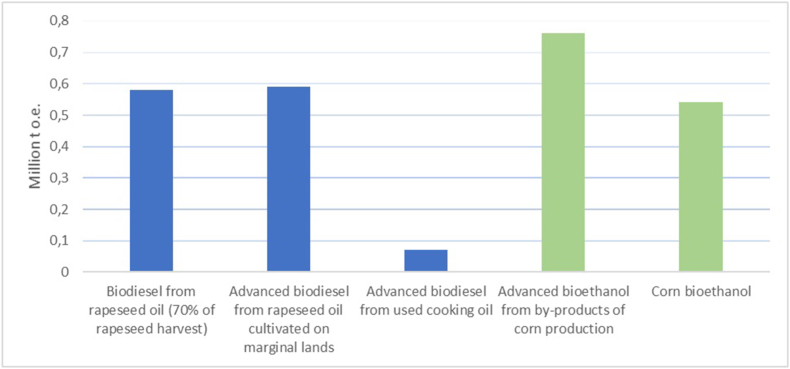
The potential of liquid biofuels consumption and output in Ukraine in 2050 [24].

According to the Bioenergy Association of Ukraine, the potential for liquid biofuels in Ukraine by 2050 is estimated at 2.54 million t o.e. (Fig. 2). The most promising feedstock for advanced biofuels in Ukraine would include the by-products of corn and oil plants growing on marginal lands [24].

UNECE and FAO estimate the potential for ethanol production in Ukraine to grow significantly by 2035, reaching 555 thousand tonnes (350 thousand t o.e.), requiring EUR 0.87 billion in investments. By 2050, this growth is expected to continue, with EUR 3.9 billion in investments needed. The potential for biodiesel production is also set to increase, estimated at 322 thousand tonnes by 2035, requiring EUR 0.62 billion in investments, and by 2050 this figure is expected to reach EUR 2.3 billion [25].

## 3 Materials and methods

To conduct the study, the following steps were undertaken:

*Formulation of research questions.* They are the estimation of the potential demand for liquid biofuel volume in Ukraine and the definition of the optimal size of the excise tax for the additive of biological origin in bioethanol. To estimate the potential demand for biofuels, we assessed the total fuel consumption and the blending mandate requirement. The first involved analyzing the national consumption of gasoline to understand current fuel usage and to determine the baseline for how much fuel is consumed. The second included calculating the amount of fuel's biological component required based on actual or potential mandatory blending regulations.

The optimal size of the excise tax was assessed using formula (1) [26]:

(1) $$A_{bio}\leq \left(C_{c}+A_{c}\right)*\lfloor \alpha \frac{\rho_{1}*Q_{1}}{\rho *Q}+\left(1-\alpha \right)\rfloor -\left[\alpha *c_{bio}+\left(1-\alpha \right)*C_{c}\right]$$

where A_bio_, A_C_ – excise tax for the biofuel and conventional fuel, respectively; C_bio_, C_c_ – price of biofuel and conventional fuel, respectively; ρ_1_, ρ – density of conventional fuel and biological component, respectively; Q_1_, Q – calorific value of conventional fuel and biological component, respectively; α – the share of biological component.

To make the necessary calculations, the following assumptions were used.

• The price of ethanol in Ukraine is UAH 34/L; the price of gasoline is UAH 62/L as of 2024;
• The density of ethanol is 0.78945 g/cm^3^; density of gasoline is 0.76 g/cm^3^ [27];
• The calorific value of gasoline - 43.6 MJ/kg, of ethanol is 30 MJ/kg;
• Gasoline consumption in Ukraine in 2020 reached 1,690.5 thousand tonnes [28]. More recent statistical data are collected but not publicly available due to martial law [29]; thus, for calculations, we assume that the approximate consumption volumes will remain the same in the coming years;
• When using bioethanol, the octane number of gasoline increases, but the distance the vehicle can cover decreases. On the E10 blend, the distance is reduced by 3 % [30], so we assume that on the E5, the distance is reduced by 1.5 %. Accordingly, 1.5 % more gasoline and ethanol will be needed to cover the same distance.
• Calorific value of diesel fuel – 43.12 MJ/kg, of biodiesel – 39 MJ/kg [31];
• The diesel fuel consumption by road transport in 2020 was 5156 thousand tonnes [28]. Due to the lack of more recent statistics, we assume that the approximate volumes of diesel fuel consumption will remain close to this value in the coming years.

*Data collection*. The data was collected using official statistics information and news on the Internet. The closure of certain types of official statistical information due to martial law explains this selection of data sources.

*Data analysis* and *policy recommendations* were formulated based on the drawbacks detected within the study.

## 4 Results

### 4.1 Legislative framework for the operation of the liquid biofuels market in Ukraine

In 2024, the Law "On Amendments to Certain Legislative Acts of Ukraine Regarding the Mandatory Use of Liquid Biofuels (Biocomponents) in the Transport Sector" [32] was adopted. The law stipulates the mandatory blending of ethanol into gasoline at a volume of 5 % starting from June 2025, and it does not provide for the blending of biodiesel into diesel fuel. The blending of biocomponents is required for gasoline with an octane number of up to 98 and below (essentially referring to A-95 gasoline), excluding gasoline for the needs of the Ministry of Defense of Ukraine and the State Reserve. The accounting for the volumes of blended biocomponents is entrusted to the State Energy Efficiency Agency. In the authors' opinion, the introduction of the blending mandate requirement is an effective measure to create an internal demand and internal market for bioethanol in Ukraine. Besides, the blending mandate requirement is the most widespread policy measure for biofuels globally (market-pull policy measure) [33]. Table 1 presents the excise tax rates for different types of fuels in Ukraine, active as of July 2024.

**Table 1:** The excise tax rates on certain types of fuel for transport in Ukraine, EUR/1000 L [34].

Fuel	Excise tax rate, EUR/1000 L
Gasoline engines with a lead content of 0.013 g/L or less (containing at least 5 % by mass of bioethanol or at least 5 % by mass of ETBE or their mixtures)	213,5
Other gasolines	213,5
Other petroleum products with a lead content of more than 0.013 g/L	213,5
Alternative transport fuel (with 30 % or more of components of biological origin)	162
Biodiesel and its mixtures (with less than 70 % by mass of petroleum or bituminous materials) based on mono-alkyl esters of fatty acids	106
Diesel fuel	213,5

The Tax Code of Ukraine anticipates that until 2035, operations related to the importation of new equipment and its component parts into Ukraine's customs territory under the customs import regime are exempt from Value Added Tax (VAT).

As of 2024, decisions are being made in Ukraine that are expected to complicate the export of grain and oilseed crops. In particular, in 2024, the Law on Amendments to the Tax Code of Ukraine and Other Legislative Acts of Ukraine Regarding the Improvement of Foreign Economic Operations for the Export of Certain Goods during Martial Law was adopted [35]. The innovations provided by the new law are expected to complicate export operations for all market participants. Specifically, the law grants the Cabinet of Ministers of Ukraine the right to establish an export security regime for grain and oilseed crops. The export of these goods will be allowed only if their price is not lower than the minimum permissible export prices (to be set by the Ministry of Agrarian Policy and Food of Ukraine) and the exporter is a VAT payer. Separate VAT regulations are provided for exporters of these goods (separate tax invoices, additional requirements for the invoices themselves). A zero VAT rate on exports will apply only if the unpaid foreign exchange earnings do not exceed 20 % of the total amount of the taxpayer's export transactions. Suppose it exceeds 20 % of the total amount of the taxpayer's export transactions. In that case, a VAT rate of 20 % or 14 % (if provided for this type of goods) will apply, and the zero rate can be used only after the submission of an adjustment calculation and the completion of currency supervision of export operations.

### 4.2 The EU feedstock requirements

Ukraine, as the Energy Community party and a candidate country for EU membership, is mandated to implement a number of EU Directives defined in the Association Agreement, including Directive (EU) 2018/2001 of the European Parliament and of the Council of December 11, 2018 on the promotion of the use of energy from renewable sources (RED II) [36]. Among other things, this Directive establishes limits on greenhouse gas emission reductions when using different types of liquid biofuels. RED II defines a number of sustainability criteria for feedstock and greenhouse gas emissions that must be met by liquids used in transport to be counted towards the overall 14 % target and to qualify for financial support from the state (Table 2).

**Table 2:** Maximum greenhouse gas emission values in RED II [36].

Start of unit/plant operation	Liquid biofuels	Liquid biofuels of non-biological origin
Before Oct 2015	50 %	–
From Oct 2015	60 %	–
From Jan 2021	65 %	70 %
From Jan 2026	65 %	70 %

Such a structured approach ensures that Ukraine aligns its renewable energy practices with EU standards and promises a future of sustainability and environmental responsibility in the country. Additionally, requirements for the consumption of advanced biofuels are established. Currently, agricultural residues (straw, husks, corn), herbaceous crops [24], sugar beet pomace, and molasses could be used as feedstock for biofuels in Ukraine. As for processed cooking oil collection, such practices are rare in Ukraine, most likely due to adverse economic feasibility. Within the 14 % transport target, there is a specific sub-target for advanced biofuels: at least 0.2 % in 2022, at least 1 % in 2025, and at least 3.5 % in 2030.

First-generation biofuels find it difficult to achieve significant greenhouse gas emission reductions, so Directive RED II imposes a 7 % cap on them and requires their phase-out by 2030.

To avoid indirect land use change (ILUC) that releases CO_2_ due to the need to produce biofuels, sustainability criteria for feedstocks and limit values for biofuels from various feedstocks are established. Biofuels that do not allow for CO_2_ emission reductions (1st generation) can still be imported and used, but they will not count towards the renewable energy consumption target in transport. Biofuel feedstocks are divided into high and low-ILUC-risk fuels [37].

The share of biofuels and bioliquids made from food and feed crops must not exceed the corresponding share of such fuels in a country's final energy consumption in the transport sector by more than one percentage point. Additionally, this share is capped at a maximum of 7 % of the final energy consumption in the automotive and rail transport sectors in any EU member state. Fuel produced from high ILUC-risk feedstocks is subject to restrictions. From 2024 to the end of 2030, this limit will gradually reduce to 0 %. Advanced biofuels will be counted with a coefficient of 1.2. For Ukraine, it means that the share of the 1st generation biofuels in the transport sector should not exceed 1.85 % to be accounted for in the quota. In contrast, the remainder of the 1st generation biofuels will not be accounted for the quota [26].

### 4.3 The prerequisites for liquid biofuels output in Ukraine

In 2020, the share of renewables in the transport sector of Ukraine was only 2.47 %, compared to the planned target of 10 % [38]. More recent statistical data have been collected but are not publicly available due to martial law. As of 2024, the indicative target for RES consumption in transport is 14 % by 2030, and there is a target of reaching not less than 27 % of energy from renewable sources by 2030, and the binding target of climate neutrality for the energy sector until 2050 [39].

As of 2024, Ukraine has many factors that determine the need for motor biofuel.

• the need to substitute the imported petroleum products;
• the availability of technological and production capacity for producing biofuels;
• the abundance of feedstock, which has significantly expanded due to the inability to export grain fully;
• commitments under the Energy Community and the Association Agreement between Ukraine and the EU, particularly the need to comply with directives promoting renewable energy use in transport.

With the start of the full-scale invasion, when the enemy destroyed Ukrainian oil refineries, Ukraine became a net importer of petroleum products. Before the full-scale invasion, petroleum products were one of the most significant import items for Ukraine. Specifically, more than half of the consumed gasoline, 80 % of gas consumption (propane-butane), and 90 % of diesel fuel were imported. In 2019, petroleum product imports amounted to 8.5 million tonnes, worth USD 5.32 billion [40].

Practically since the beginning of the full-scale invasion, Ukraine's seaports, which predominantly exported grain from Ukraine, have been blocked by the enemy's fleet and armed forces. As a result, Ukraine temporarily lost the ability to export grain via maritime routes. Before the full-scale invasion, Ukraine exported 45–60 million tons of grain annually (for example, in the 2020/2021 marketing year, Ukraine exported 45.7 million tonnes of grain, of which wheat accounted for 17.5 million tons, and corn – 23.5 million tons). During the blockade of the Black Sea, thanks to the Istanbul grain agreement of July 22, 2022, Ukraine managed to export 32.8 million tonnes of grains and oilseeds. The agreement initially provided for the export of up to 25 million tonnes of grain; however, during its implementation, the terms of the agreement and its (limited) duration offered numerous opportunities for manipulation by the enemy, which created significant uncertainties for Ukrainian grain traders, and led to higher freight rates in the ports of Reni, Kiliya, and Izmail [41]. The port blockade resulted in economic losses for agricultural producers amounting to approximately USD 15 billion in the first year of the full-scale invasion [42]. Despite these challenges, Ukraine's agricultural sector has shown remarkable resilience, with grain exports from Ukraine in the 2022/2023 marketing years (i.e., fully wartime years) even higher than in the 2021/2022 marketing year. In the 2022/2023 marketing year, 48.4 million tonnes were exported. This was made possible by increased exports of wheat and barley to Turkey and significant growth in exports to Romania and Spain. Corn exports increased from 23 million tonnes to 29 million tonnes. Overall, two-thirds of the food was exported through the so-called "grain corridor".

At the same time, several EU countries (Poland, Hungary, Bulgaria, Slovakia, Romania) banned the export of Ukrainian grain to their countries. Poland even prohibited the transit of grain through its territory. However, grain and oilseeds from Ukraine accounted for only 5 % of Poland's production and no more than 10 % of the European market [42]. In the ranking of grain buyers from Ukraine, Poland was fifth for grain, eighth for barley, and seventh for corn but first for the volume of rapeseed purchases. The analysis of this ban's appropriateness lies outside this study's scope and primarily aims to protect European agricultural producers. However, the limited maritime export of food and grain supply via EU countries cause a grain surplus in Ukraine. Overall, the annual throughput capacity of the EU's land points is 12 million tonnes per year, while Ukraine is ready to ship up to 84 million tonnes per year [43]. The 2024 forecast for grain output is 56 million tonnes, and for oil crops – 21 million tonnes, as estimated by the Ministry of Agrarian Policy and Food of Ukraine [44].

Since the storage capacity for grain is limited, the surplus grain needs to be processed domestically. The production of even first-generation biofuels is a solution to the problem. Given feedstock availability, the abundant 23 million tonnes of grain, even with a low starch content (60 %), could produce 8.28 billion L of ethanol (assuming that 1 tonne of grain with 60 % starch content yields 360 L. Typically, this applies to wheat and barley, whereas corn usually contains 70 % starch, which can yield 420 L of ethanol [45]). With an ethanol density of 0.783 kg/m³, 8.28 billion L can be converted into 6.48 million tonnes of ethanol. In terms of raw material supply, Ukraine has 3.5 times more feedstock for bioethanol production than it can consume in gasoline (in volumetric terms). Considering the production capacities, Ukraine can already provide an E10 blend with the prospect of increasing the blend to E30 and higher, which could lead to a significant reduction in tailpipe CO_2_ emissions [46].

As of Aug 2023, the cost of 96 % ethanol for industrial production was 24–25 UAH/L, which is half the retail price of gasoline. The cost of corn from grain traders on the domestic market as of August 2024 was 5.6–7 thousand UAH/tonne [47], and wheat was 5.411 thousand UAH/tonne [48]. Thus, the feedstock component of the biofuel component from 1 tonne of corn was 13.3–16.7 UAH/L, and from wheat - 15 UAH/L.

As of Jun 2024, the cost of ethanol on the international market is 700 USD/m^3^, gasoline - 700–800 USD/m^3^; the cost of diesel fuel - 800–1000 USD/m^3^, and biodiesel - 2000 USD/m^3^ [49].

The feedstock for the 1st generation biodiesel (primarily rapeseed) was almost entirely export-oriented even before the full-scale invasion: on average, 91 % of rapeseed produced in Ukraine is exported [41,50]. Ukraine is among the world's top five rapeseed exporters (after Canada, the EU, China, and Australia) [51]. In 2021, rapeseed exports amounted to USD 1.3 billion [52], while in 2022 – USD 1.6 billion [53]. Rapeseed oil is a much more common food product in many European countries than sunflower oil. Foreign counterparts continue to show significant interest in Ukrainian products because of their value characteristics and geographical proximity to Ukraine and because Ukrainian rapeseed is not genetically modified [34]. The main export destinations are predominantly EU countries, such as Germany, Belgium, France, the Netherlands, Portugal, etc.

The full-scale invasion of Ukraine has had a significant impact on the country's export volumes. Since the beginning of the Russian invasion and the blockade of Ukrainian Black Sea ports, export volumes have been noticeably restricted. For example, Ukraine typically and regularly exports millions of tonnes of bulk goods such as sunflower seeds, rapeseed, and wheat. However, since the beginning of the full-scale invasion, exports have been sporadic. Despite the significantly smaller volumes, Ukrainian traders have managed to find alternative routes and supply goods to Bulgaria, Turkey, Lithuania, Latvia, Romania, and Germany. Over the next 3–5 years, rapeseed prices are expected to increase by more than 3 % annually due to growing demand for rapeseed and increased industrial consumption, including transportation needs. In 2022, Ukraine managed to produce 3.5 million tonnes of rapeseed, which is even more than in 2021 (2.96 million tonnes).

Kyiv School of Economics forecasts that the significant export orientation of rapeseed will remain in the long term, while the export of soybeans will be negligible. Even in 2022–2023, the areas to produce rapeseed were growing because rapeseed allows much higher incomes than other coil crops in Ukraine. The internal demand for rapeseed may increase by 2033 due to the growing poultry output (where rapeseed cake is used as fodder) [50]. It creates additional competition with biofuels in general, where biofuels lose.

### 4.4 The capacity of the liquid biofuels market in Ukraine

**Fig. 3:**
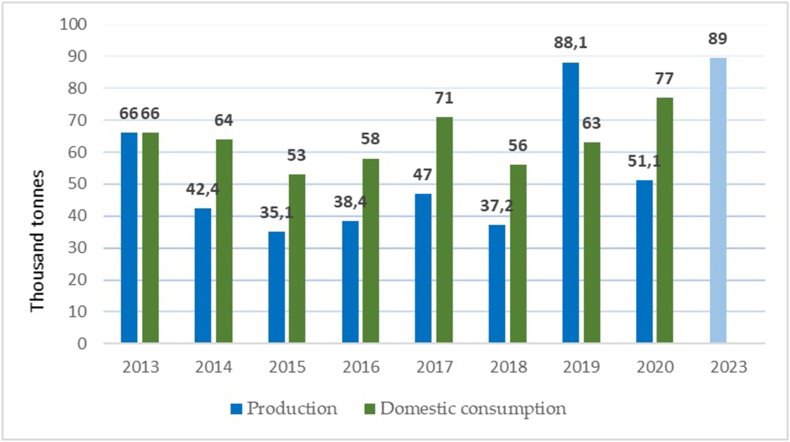
Volumes of Biofuel Consumption in Ukraine, 2013–2020, thousand tonnes [25,41].

Below, we consider the market capacity for the liquid biofuels in Ukraine. According to the Law, blending 5 % bio-component with gasoline defines the market capacity at a minimum of 80 thousand tonnes of ethanol, requiring about 1 million tonnes of corn [50]. For more detailed calculations, we use pre-invasion data for Ukraine (since newer data is unavailable). The actual volumes of biofuel consumption in Ukraine in 2013–2020 are shown in Fig. 3.

Fig. 3 above depicts mainly ethanol consumption, not diesel fuel with blended biological components. Fourteen large biodiesel plants with an installed capacity of 300,000 t/year and about 50 small biodiesel plants with a capacity of 25,000 t/year used to operate in Ukraine. Due to the industry's overregulation (in particular, the high excise tax), this business lost profitability, so the biodiesel production capacity was mainly converted to the production of dyes.

To determine the capacity of the liquid biofuel market of Ukraine, we calculate the potential volumes of consumption of liquid biofuels, separately for bioethanol and biodiesel. It should be noted that we can use only data and observations from the pre-invasion period for further calculations. During the full-scale invasion period, the numbers are different due to the decline in the purchasing power of citizens, unemployment, and large volumes of migration abroad, including on their own vehicles. Calculations may be changed as updated statistical observations are made public. We assume a certain minimum proportion of ethanol must be mixed with gasoline. We will estimate the amount of replacement of fossil fuel (gasoline) under the condition of ethanol admixture in Ukraine in the amount of 5 % by volume with a gradual increase in the content of the biological component of the fuel up to 11 % (Table 3).

**Table 3:** The amount of bioethanol needed to substitute imported fuel, tonnes.

Year	Share of the biological component (ethanol), %	Substituted gasoline, tonnes	Biological component required to substitute fossil fuel, tonnes	Ethanol needed considering "gaining" distance, tonnes
2025	5	84525	122561	124400
2026	6	101430	147074	149280
2027	7	118335	171586	175017
2028	8	135240	196098	200020
2029	9	152145	220610	225022
2030	10	169050	245123	252476
2031	11	185955	269635	277724

As can be seen from the calculations in Table 3, to meet the needs of the Ukrainian market, subject to the introduction of the requirement for ethanol admixture in the amount of 5 % from 2025 and the increase of the latter to 11 % by 2031, 125 to 278 thousand tonnes of ethanol will be needed.

During the full-scale invasion, Ukraine became a net importer of petroleum products. It is worth noting that Ukraine is not supplied with "pure" gasoline (Eurobob) from the EU but a blend that may already contain a small amount of added biological component. The content of the biological component can be assumed to not exceed 5 %, so after the "treatment" of gasoline in Ukraine, it meets the "Euro-5″ standard. Below, we calculate the capacity of the domestic biodiesel market in Ukraine (Table 4).

**Table 4:** The amount of biodiesel needed to substitute imported fuel, tonnes.

Year	Share of the biological component, %	Substituted diesel fuel, tonnes	Biological component required to substitute fossil fuel, tonnes
2025	5	257800	284869
2026	6	309360	341843
2027	7	360920	398817
2028	8	412480	455790
2029	9	464040	512764
2030	10	515600	569738
2031	11	390170	431138

As seen from the above calculations, the approximate capacity of the Ukrainian biodiesel market can range from 196 to 431 thousand tonnes per year. Such volumes of biodiesel consumption are not realistic for many reasons (export of rapeseed, low feasibility of rapeseed biodiesel etc.).

**Fig. 4:**
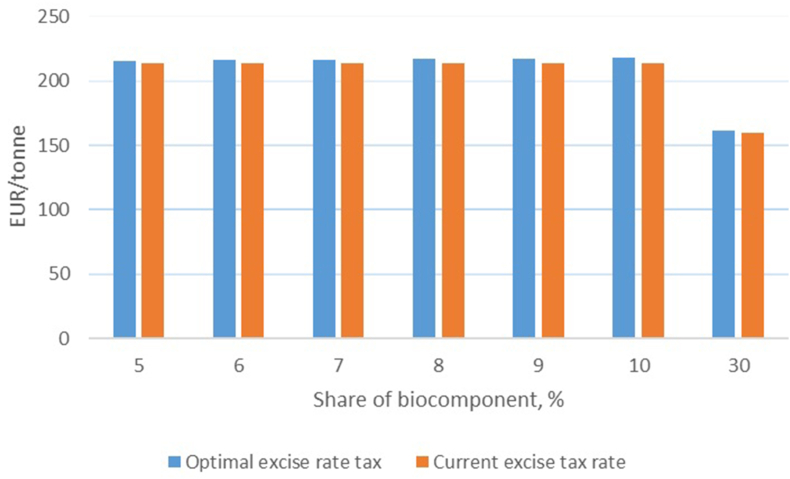
The current and optimal excise tax rates for bioethanol in Ukraine.

The rates of excise taxes for ethanol obtained are depicted on Fig. 4; there are compared to the current excise taxes rate.

The same formula could be applied to calculate the optimal excise rate for biodiesel. However, as of 2024, biodiesel is not produced or sold in Ukraine, and the lack of a price makes the calculations impossible.

## 5 Discussion

The estimated market capacity for bioethanol production in Ukraine is increasingly attainable, bolstered by the current production capacity of 187.5 thousand tonnes and ongoing expansion efforts. The launch of the Vitagro Energy bioethanol plant in 2024, with a capacity of 25 thousand tonnes per year and ISCC certification, marks a significant milestone that allows for ethanol export to European markets. Furthermore, the development landscape is promising, with at least three additional plants in the design phase, three more in commissioning, and one under construction. Notably, "OKKO", one of Ukraine's largest fuel station networks, is constructing a facility with an 85-thousand-tonne capacity. To support this growth, investment in production capacity ranges from 500 to 1,000 USDper tonne of feedstock, necessitating approximately EUR 100 million to achieve the target of 125 thousand tonnes of ethanol in Ukraine. Importantly, all new facilities under development are expected to produce advanced bioethanol.

Historically, Ukraine's internal market lacked sufficient demand, leading to a reliance on exports for its ethanol production. However, protective measures have emerged; in 2023, Poland banned not only the import of alcohol from Ukraine but also its transit [51]. As of 2024, the EU has implemented import quotas on Ukrainian ethanol. Despite this, selling ethanol above the quota remains profitable, with duties set at 100 EUR per tonne for a market price of 700 EUR per tonne [52]. The introduction of a blending mandate requirement law, effective from June 2025, is expected to generate predictable internal demand for ethanol, enhancing market stability.

In contrast, the current prices for rapeseed and rapeseed oil render rapeseed biodiesel economically unfeasible in Ukraine. Before the full-scale invasion in 2022, Ukraine enjoyed a surplus of electricity, providing a solid foundation for developing electric mobility across railways and electric vehicles. However, the destruction of electricity-generating infrastructure has resulted in a deficit, potentially shifting consumer preferences toward vehicles with internal combustion engines, thereby increasing demand for biofuels.

Ukraine possesses approximately 4 million ha of unused agricultural land [24]; a situation exacerbated by ongoing hostilities is likely to increase the area of marginal lands to another 5 million ha. Although these lands remain suitable for producing agricultural commodities, which serve as feedstock for first-generation biofuels, their classification as marginal could transition them toward advanced biofuels production.

When planning economic activities related to biofuel production, it is crucial to consider the competition for feedstock availability against other demands, particularly for biogas and biomethane. Currently, biomethane is regarded as the most promising bioenergy product in Ukraine, necessitating careful strategic planning to balance these competing interests effectively. It underscores the importance of strategic planning in the decision-making process.

To ensure the development of the biofuels market in Ukraine, the law on the mandatory blending of biological components needs to be amended to include the blending of biodiesel and other liquids of biological origin. Information campaigns on the benefits of biofuels need to be held; the vehicles using the biofuels (with 30 % of biological components) are to be marked respectively, and the users of such vehicles should have the same benefits as users of e-vehicles (permission to use advantageous parking spots, etc.

One major limitation of the study is that it is based on the fuel consumption of pre-war Ukraine. As mentioned earlier, the precise statistics on fuel consumption are not known; once they are, the assessments need to be changed.

Given the technological developments and increased requirements for advanced biofuels, future support policies have to include further support measures for them, which is a subject for further study.

## 6 Conclusions

The ongoing geopolitical challenges in Ukraine underscore the urgent need to transition to liquid biofuels as a viable solution for enhancing energy independence and meeting domestic energy demands. The country possesses substantial potential for biofuel production, estimated at 2.54 million tonnes of oil equivalent by 2050. The projected demand for bioethanol and biodiesel between 2025 and 2031 highlights significant opportunities for market growth, with estimates ranging from 125 to 278 thousand tonnes for bioethanol and 196 to 431 thousand tonnes for biodiesel. bioethanol's current excise tax rates appear reasonable and supportive of industry development.

## Ethical statement

This work does not involve the use of humans or animals.

## Data availability statement

Not applicable.

## Funding

This work research was supported by the 10.13039/501100004742National Academy of Sciences of Ukraine as part of the scientific project "Innovative Modernization of Prospective Industries in Ukraine in the Post-War Period Based on Existing Scientific, Technical, Production, and Resource Potential." Phase II: Prospects for the Creation of Innovative Industrial Sites in Ukraine for the Production of High Value-Added Products in the Context of Integration into International Production Chains (2024). (State Registration Number 0123U102325).

## Declaration of competing interest

The authors declare that they have no known competing financial interests or personal relationships that could have appeared to influence the work reported in this paper.
