# Supplementary material for: Unlocking the potential: Key factors shaping the liquid biofuels market in Ukraine
Source: Heliyon. 2024 Nov 15;10(22):e40420. doi: 10.1016/j.heliyon.2024.e40420 (PMC11617215; doi:10.1016/j.heliyon.2024.e40420)
Supplement: Multimedia component 1 [file mmc1.docx]

**Table 1**

The excise tax rates on certain types of fuel for transport in Ukraine, EUR/1000 L [34]

| Fuel | Excise tax rate, EUR/1000 L |
| --- | --- |
| Gasoline engines with a lead content of 0.013 g/L or less (containing at least 5% by mass of bioethanol or at least 5% by mass of ETBE or their mixtures) | 213,5 |
| Other gasolines | 213,5 |
| Other petroleum products with a lead content of more than 0.013 g/L | 213,5 |
| Alternative transport fuel (with 30% or more of components of biological origin) | 162 |
| Biodiesel and its mixtures (with less than 70% by mass of petroleum or bituminous materials) | 106 |
| Diesel fuel | 213,5 |

**Table 2**

Maximum greenhouse gas emission values in RED II [36]

| Start of unit/plant operation | Liquid biofuels | Liquid biofuels of non-biological origin |
| --- | --- | --- |
| Before Oct 2015 | 50% | - |
| From Oct 2015 | 60% | - |
| From Jan 2021 | 65% | 70% |
| From Jan 2026 | 65% | 70% |

**Table 3**

The amount of bioethanol needed to substitute imported fuel, tonnes

| Year | Share of the biological component (ethanol), % | Substituted gasoline, tonnes | Biological component required to substitute fossil fuel, tonnes | Ethanol needed considering "gaining" distance, tonnes |
| --- | --- | --- | --- | --- |
| 2025 | 5 | 84525 | 122561 | 124400 |
| 2026 | 6 | 101430 | 147074 | 149280 |
| 2027 | 7 | 118335 | 171586 | 175017 |
| 2028 | 8 | 135240 | 196098 | 200020 |
| 2029 | 9 | 152145 | 220610 | 225022 |
| 2030 | 10 | 169050 | 245123 | 252476 |
| 2031 | 11 | 185955 | 269635 | 277724 |

**Table 4**

The amount of biodiesel needed to substitute imported fuel, tonnes

| Year | Share of the biological component, % | Substituted diesel fuel, tonnes | Biological component required to substitute fossil fuel, tonnes |
| --- | --- | --- | --- |
| 2025 | 5 | 257800 | 284869 |
| 2026 | 6 | 309360 | 341843 |
| 2027 | 7 | 360920 | 398817 |
| 2028 | 8 | 412480 | 455790 |
| 2029 | 9 | 464040 | 512764 |
| 2030 | 10 | 515600 | 569738 |
| 2031 | 11 | 390170 | 431138 |
