# Supplementary material for: Unlocking the potential: Key factors shaping the liquid biofuels market in Ukraine
Source: Heliyon. 2024 Nov 15;10(22):e40420. doi: 10.1016/j.heliyon.2024.e40420 (PMC11617215; doi:10.1016/j.heliyon.2024.e40420)
Supplement: Multimedia component 2 [file mmc2.docx]

$A_{bio}\leq\left( C_{c}+A_{c} \right)*\left\lfloor\alpha\frac{\rho_{1}*Q_{1}}{\rho*Q}+\left( 1-\alpha\right) \right\rfloor- \left[ \alpha*c_{bio}+\left( 1-\alpha\right)*C_{c} \right]$ (1),

where A_bio_, A_C_ – excise tax for the biofuel and conventional fuel, respectively; C_bio_, C_c_ – price of biofuel and conventional fuel, respectively; ρ_1_, ρ – density of conventional fuel and biological component, respectively; Q_1_, Q – calorific value of conventional fuel and biological component, respectively; α – the share of biological component.
